# Base resolution maps reveal the importance of 5-hydroxymethylcytosine in a human glioblastoma

**DOI:** 10.1038/s41525-017-0007-6

**Published:** 2017-03-13

**Authors:** Eun-Ang Raiber, Dario Beraldi, Sergio Martínez Cuesta, Gordon R. McInroy, Zoya Kingsbury, Jennifer Becq, Terena James, Margarida Lopes, Kieren Allinson, Sarah Field, Sean Humphray, Thomas Santarius, Colin Watts, David Bentley, Shankar Balasubramanian

**Affiliations:** 1 Cancer Research UK Cambridge Institute, University of Cambridge, Cambridge, UK; 2 Department of Chemistry, University of Cambridge, Cambridge, UK; 3 Illumina Ltd., Chesterford Research Park, Little Chesterford, Saffron Walden, UK; 4 Department of Pathology, Addenbrooke’s Hospital, Cambridge University Hospitals, Cambridge, UK; 5 Department of Clinical Neurosciences, Division of Neurosurgery, University of Cambridge, Cambridge, UK; 6 School of Clinical Medicine, University of Cambridge, Cambridge, UK

## Abstract

Aberrant genetic and epigenetic variations drive malignant transformation and are hallmarks of cancer. Using PCR-free sample preparation we achieved the first in-depth whole genome (hydroxyl)-methylcytosine, single-base-resolution maps from a glioblastoma tumour/margin sample of a patient. Our data provide new insights into how genetic and epigenetic variations are interrelated. In the tumour, global hypermethylation with a depletion of 5-hydroxymethylcytosine was observed. The majority of single nucleotide variations were identified as cytosine-to-thymine deamination products within CpG context, where cytosine was preferentially methylated in the margin. Notably, we observe that cells neighbouring tumour cells display epigenetic alterations characteristic of the tumour itself although genetically they appear “normal”. This shows the potential transfer of epigenetic information between cells that contributes to the intratumour heterogeneity of glioblastoma. Together, our reference (epi)-genome provides a human model system for future studies that aim to explore the link between genetic and epigenetic variations in cancer progression.

## Introduction

Genetic and epigenetic alterations to the genome shape the development of human malignancies. The patterns of the DNA methylation mark 5-methylcytosine (5mC) become aberrant in human malignancies and affect cellular functions.^1^ The recently re-discovered DNA mark 5-hydroxymethylcytosine (5hmC)^2, 3^ is a functionally important DNA modification, and is an intermediate in the process of active demethylation of 5mC. In cancers, 5hmC patterns undergo considerable changes^4^ that have been linked to genome instability^5, 6^ and remodelling of the DNA methylation pattern.^7^ Previous studies revealed that 5hmC is consistently found at significantly reduced levels in various solid tumours.^8–10^ Indeed, epigenetic regulators such as DNA methyltransferases (DNMT), ten-eleven-translocation (TET) proteins or isocitrate dehydrogenases (IDH), are crucial for normal and malignant cellular developement.^11^ Very few studies however, have effectively mapped the distribution of 5hmC in normal or cancer tissues. Herein, we present the first single base resolution maps of whole genomes, methylomes, and hydroxymethylomes for matched human glioblastoma and tumour margin samples.

## Results

### Enhanced (hydroxyl)-methylome sequencing reveals global hypermethylation in tumour with loss of 5hmC

**Fig. 1:**
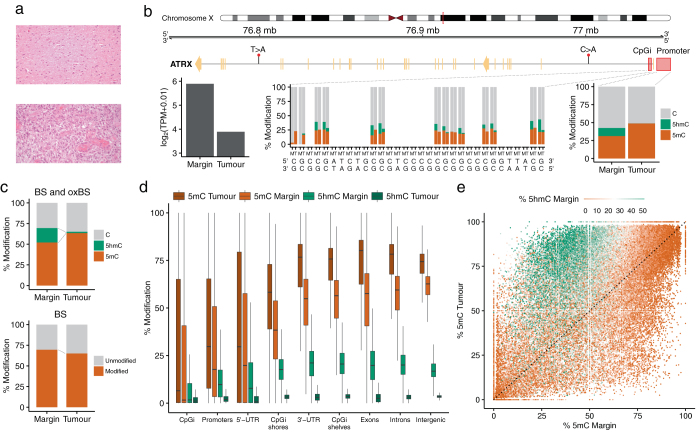
Cytosine modification landscape of all CpG sites (*n* = 2.7 × 10^7^) in a glioblastoma patient. **a** Hematoxylin and eosin (H&E) staining of the margin (*top*) and tumour (*bottom*) samples (see Methods). **b** Base resolution outlook of 5mC and 5hmC levels, nucleotide alterations and changes in transcript levels between margin (M) and tumour (T) in the *ATRX* gene. The close view highlights 32 bp of the CpG island (chrX:77041003-77041725) located in the 5′-UTR region (*middle*) and average levels of modification across the 1 kb promoter (chrX:77041719-77042719) (*right*). Transcript levels (*left*) are displayed in transcripts per million (TPM). **c** Combination of BS and oxBS-seq reveals a severe decrease of 5hmC in tumour accompanied by an increase of 5mC, however BS-seq only cannot distinguish between 5mC and 5hmC, and would suggest tumour hypomethylation. Distribution of 5mC and 5hmC in the margin and tumour samples **d** across genomic regions and **e** in a sample of 10^5^ CpG sites colour-coded according to the level of 5hmC in the margin

We performed whole genome sequencing at 100× coverage of blood, tumour, and margin samples from a glioblastoma patient (Fig. 1a) using a PCR-free library preparation.^12^ Total RNA sequencing of all three samples was also performed. We employed oxidative bisulfite sequencing (oxBS-seq)^13^ and bisulfite sequencing (BS-seq) to generate high-depth (80×) sequence coverage and built single-base resolution maps that distinguished 5mC and 5hmC modifications (Fig. 1b and Supplementary Tables 1 and 2). In the margin sample, we found levels of 50% for 5mC and 20% for 5hmC integrated over all CpGs in the genome, whereas in the tumour, we observed global hypermethylation, with average levels of 60% 5mC and a drastic loss of 5hmC to 1.6% (Fig. 1c).

We then assessed specific regions within the genome that were differentially methylated between tumour and margin and identified substantial hypermethylation in the 3′-UTRs, CpGi shelves, exons and introns of the tumour compared to the margin (Fig. 1d). We observed that CpGs with high levels of 5hmC in margin tissue have correspondingly higher levels of 5mC in tumour (Fig. 1e) suggesting that elevated 5mC sites in tumour DNA have arisen through failure to oxidise 5mC to 5hmC.

### Gene promoter (hydroxyl)-methylation and transcript levels

**Fig. 2:**
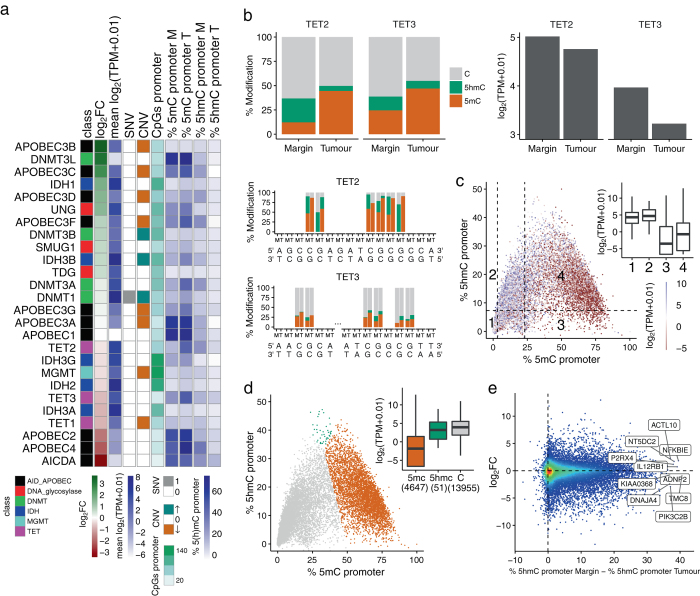
Overview of the relationship between genetic changes, promoter 5mC/5hmC levels and gene expression. **a** Summary of the molecular details of the genes involved in the turnover of cytosine modifications. Differential transcript levels between tumour and margin (log_2_FC where FC = (TPM_tumour_ + 0.01)/(TPM_margin_ + 0.01)) and mean transcript levels ((log_2_(TPM_tumour_ + 0.01) + (log_2_(TPM_margin_ + 0.01))/2), SNVs (1: presence and 0: absence), CNVs (↑: gain of copies, 0: diploid and ↓: loss of copies and LOH: loss of heterozygosity), promoter CpG counts, and promoter 5mC and 5hmC levels (%) in margin (M) and tumour (T), which are colour-coded as shown in the legend. Genes bearing genomic alterations in glioma progression^24^ were also examined (Supplementary Fig. 1). **b** Average and base resolution maps of 5mC and 5hmC levels and changes in transcript abundance between margin and tumour in the promoter region (1 kb upstream of the transcription start site) of the *TET2* (chr4:106066031-106067031, 22 CpG sites) and *TET3* (chr2:74272449-74273449, 6 CpG sites) genes. **c** Cytosine modifications and changes in transcript levels across all gene promoters containing CpG sites (*n* = 18,653) in margin. Promoters are divided into four sectors according to the levels of 5mC and 5hmC: sectors {1, 2} and {3, 4} contain low and high levels of 5mC according to the first and third terciles (3.2 and 23.2%) of the %5mC distribution respectively (*horizontal axis*). The median level of 5hmC (7.4%) is used to separate low and high 5hmC levels (*vertical axis*). The inset box plot displays the transcript levels for each sector. **d** Promoters are divided into three types depending on whether 5mC, 5hmC or C is more abundant within the promoter. The *inset box plot* illustrates the transcript levels for each promoter type. **e** Relationship between differential transcript levels (log_2_FC) and differences in 5hmC levels between tumour and margin in gene promoters containing more than 10 CpG sites (*n* = 15,716). The top ten promoters with larger changes in 5hmC levels are labelled

To investigate the possible mechanism that could account for the loss of 5hmC, we looked for loss-of-function mutations or epimutations and identified single base resolution (hydroxy)-methylation changes in the promoter in connection to transcript levels of key epigenetic regulators (Fig. 2a). Apparent loss of 5hmC in tumours can occur through loss-of-function mutations in *TET* enzymes that oxidise 5mC to 5hmC, or inhibition of *TET* activity by the oncometabolite beta-hydroxyglutarate generated by mutant *IDH1/2*.^14–17^ *IDH* mutations are mutually exclusive with mutations in *TET*, at least in acute myeloid leukaemia.^18^ No *IDH* mutations or loss-of-function mutations in the *TET* genes were observed in the tumour DNA for this patient. However, we observed hypermethylation at *TET2/3* gene promoters with concomitant loss of 5hmC at the same CpGs and a corresponding reduction in *TET2/3* expression in the tumour (Fig. 2b). *TET2* promoter methylation has previously been observed in low-grade diffuse gliomas lacking *IDH1/2* mutations and provides a third mechanism to cause loss in maintenance of 5hmC levels in the tumour. Previous literature has also linked reduced *TET* function to solid and myeloid malignancies^5, 19, 20^ and suggested a key role for *TET* in the prevention of cancer by suppressing cell invasion^21^ and promoting genome integrity.^5, 6^ Our results in the current glioblastoma case are consistent with these ideas.

Transcript levels in margin are related to the levels of both, methylation and hydroxymethylation in promoters (Fig. 2c, d). The transcript levels are low at genes whose promoters have a high level of 5mC but a low level of 5hmC (sector 3, Fig. 2c). When 5hmC is high and 5mC is low (sector 2, Fig. 2c) transcript levels are high. This fits a model where 5mC marks silent promoters, while 5hmC and C mark active or poised promoters (Fig. 2d).^22^ Interestingly, even when 5mC is high and 5hmC levels shift from low to high (sectors 3 to 4, Fig. 2c), the expression is increased. Quantitative analysis of mRNA levels revealed that 8141 genes (24%) were differentially expressed (logFC > 2 or logFC < −2) between the margin and tumour (Supplementary Fig 2) with Fig. 2e highlighting expression levels of the top ten most differentially hydroxymethylated promoters.

### Patterns of genomic variations

**Fig. 3:**
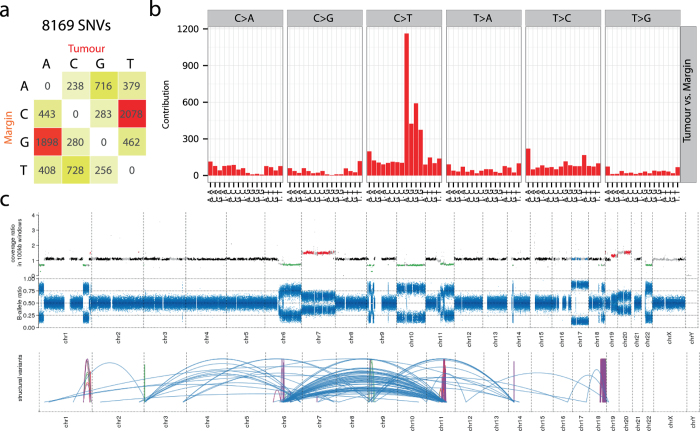
Genetic variation landscape of the tumour sample. **a** 8169 somatic variants, mostly C to T transitions, identified when comparing the tumour and margin samples. **b** The 5′ and 3′ nucleotide context around the somatic variants in tumour suggests that most mutations occur in CpG sites. **c** At the top, genomic location of CNVs: polyploid regions (Gain) in *red*, diploid regions (Ref) in *black*, haploid regions (Loss) in *green*, and loss of heterozygosity (LOH) in *blue*. In the middle, B-allele ratio plot. At the bottom, genomic translocations (*blue*) and inversions (*purple*) in the tumour

We analysed the mutational landscape by identifying single nucleotide variants (SNVs) in tumour and margin using the blood sample as a reference. Although we identified 8169 SNVs in the tumour (Fig. 3a), the margin appeared genetically normal when compared to blood (Supplementary Fig. 3). About 50% of all SNVs in the tumour were C to T changes (or G to A, opposite strand), most often in NpCpG contexts, corresponding to the mutational signature 1A as ﻿described in Alexandrov et al. (Fig. 3b), though we did not observe any *kataegis* formation.^23^ SNVs identified within the coding region of several cancer genes suggest aberrations of the *RTK–RAS–PI3K* signalling (mutations in *PTEN*, *PIK3R6*, and *NF1*) and *MYC* signalling pathways (*SMARCB1* and *LZTR1*), and have been recently described in a genomic characterisation of *IDH*(+) glioma patients.^24^ Additionally, complex patterns of somatic structural variants were characteristic of the tumour (Fig. 3c). We observed many translocation and inversion events with additional copy number gains in chromosomes 7, 17, and 20, and copy number losses in chromosomes 1, 6, 9, 10, 11, 18, and 22. Notably, the epigenetic modifiers *TET1* (chromosome 10) and *DNMT1/3B* (chromosomes 19 and 20) were haploid and polyploid in tumour respectively, which suggests a connection between the observed hypermethylation (*DNMT* polyploidy) and 5hmC loss (diminished *TET* function) in the tumour.

### Links between genomic and epigenomic changes

**Fig. 4:**
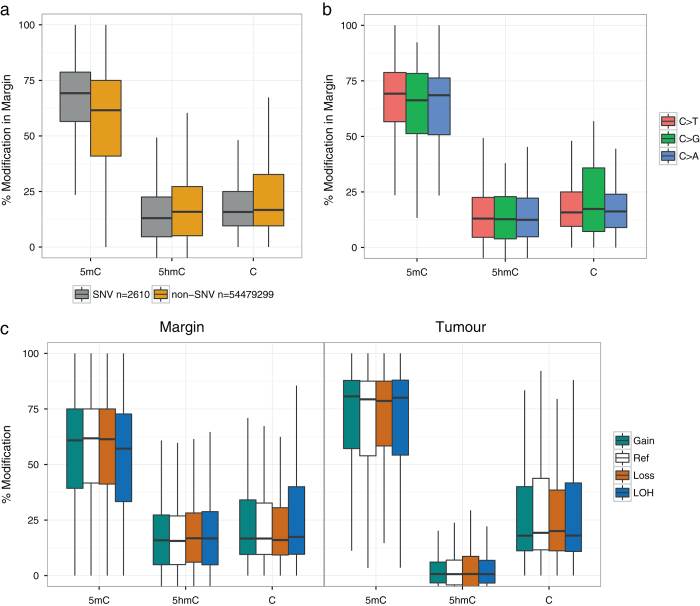
Link between genetic variation and cytosine modifications. **a** Analysis of 5mC and 5hmC levels within the margin CpG sites that are mutated in tumour (SNVs). **b** Differences in modification levels depending on the mutated base in tumour: C>T (*n* = 2527), C>G (*n* = 32) and C>A (*n* = 51). **c** Distribution of modification levels in margin and tumour in regions with gain or loss of copies, diploid (Ref) or loss of heterozygosity (LOH) according to the CNV analysis of the tumour sample (Fig. 3c)

To investigate links between genetic and epigenetic mutations, we identified SNV sites resulting from the mutation of cytosine to any other base in the tumour, and studied their methylation and hydroxymethylation status in the margin (Fig. 4a). We found that SNV sites were significantly more methylated in the margin compared to non-SNV sites (Mann–Whitney test, *p*-value < 2.2e–16, two-sided). Conversely, we found that SNV sites were significantly less hydroxymethylated in the margin compared to non-SNV sites (Mann–Whitney test, *p*-value < 2.2e–16, two-sided) with no apparent differences for all types of cytosine mutations (i.e. C to, C to G, or C to A) (Fig. 4b). Regions identified to be of different ploidy in margin and tumour (Fig. 3c) did not show differences in modification levels (Fig. 4c).

Tumour purity was estimated to be 71% by genetic analysis and 60% by immunohistochemistry (see Methods). Based on tumour purity, we calculated the expected global level of 5hmC to have a lower limit of 7% (Table 1), assuming complete loss of 5hmC in the tumour. We measured actual levels of 5hmC in the tumour sample to be 1.6%, more than four-fold lower than the estimated lower limit.

**Table 1:** Overall cytosine modification levels of all CpG sites (*n* = 2.7 × 10^7^) in a glioblastoma patient

Sample	% 5mC	% 5hmC
Margin	52.1	17.5
Tumour	63.5	1.6

## Discussion

We used a PCR-free approach to generate accurate methylomes and hydroxymethylomes. Importantly, our ability to resolve 5mC and 5hmC signals reveals global hypermethylation in the tumour, contrary to the appearance of global hypomethylation when using only BS-seq, which actually measures the sum of 5mC and 5hmC and fails to resolve the two signals. Global hypermethylation has just recently also been reported for kidney cancer using an alternative method that was able to distinguish 5mC from 5hmC.^25^ While genome-wide hypomethylation^26, 27^ is somewhat regarded as an epigenetic hallmark of tumorigenesis and has been implicated in the evolution of human glioblastomas,^28^ our results suggest that data obtained from bisulfite-only approaches need to be re-interpreted.

*TET2* promoter methylation has previously been observed in low-grade diffuse gliomas lacking *IDH1/2* mutations and provides a third mechanism to cause loss in maintenance of 5hmC levels in the tumour. Previous literature has also linked reduced *TET* function to solid and myeloid malignancies^5, 19, 20^ and suggested a key role for *TET* in the prevention of cancer by suppressing cell invasion^21^ and promoting genome integrity.^5, 6^ Our results are consistent with this mechanism in the current glioblastoma case.

5mCpG sequences have previously been reported to be mutational hotspots in human genetic disease and cancer-relevant genes, probably through the deamination of 5mC to T.^29^ Our data show that 5mCpG sites are predisposed to mutations, whereas 5hmCpG sites are protected from mutations during tumorigenesis, mirroring observations recently reported for brain and kidney cancer, and myeloid leukaemias.^30^ Interestingly, the ‘protection’ at 5hmCpG sites holds for all types of cytosine mutations (C to T, C to G, or C to A).

Our observation that 5hmC levels in the tumour are significantly lower than the estimated lower limit suggests a loss of 5hmC in the genetically “normal” cells within the tumour mass. Earlier studies reported that genome-wide methylation changes in tumours can be acquired in adjacent normal cells linking 5mC to field defects.^31–33^ The concept of field defects^34^ has been used to describe the early events in the stepwise transformation of the cancer that can potentially lead to further oncogenic changes. To the best of our knowledge, this is the first observation of acquired, early changes in 5hmC in proximal cells.

Accurate, high resolution whole genome maps have allowed us to discern a role for 5hmC in protecting the genome against somatic mutations, observe global hypermethylation in a tumour and provide new evidence that associates 5hmC with epigenetic transformation in genetically normal cells adjacent to the tumour. Epigenetic analysis of 5hmC, in addition to 5mC, may foster diagnostic approaches in the future.

## Methods

### Clinical events

A 71-year old woman underwent supra-total resection of a right frontal intrinsic tumour with maximum diameter of the enhancing component of 19 mm, surrounded by only minimal oedema. Histological examination confirmed the diagnosis of glioblastoma multiforme (WHO 9440/3). Tissue collection protocols were compliant with the UK Human Tissue Act 2004 (HTA License ref. 12315) and approved by the Local Regional Ethics Committee (LREC ref. 04/Q0108/60). Informed consent was obtained from the patient before surgery. Surgical samples were taken from the enhancing tumour mass and corresponding non-enhancing margin using advanced surgical techniques described previously.^35^ After rapid recovery she underwent six weeks of radiotherapy (60Gy in 30 fractions) with concurrent temozolomide (TMZ) treatment. She then completed two cycles of adjuvant TMZ treatment during 12 weeks. This was stopped because of poor tolerance. While her 9-months post-operative scan was clear of tumour, her 11-months check demonstrated tumour recurrence and she died 3 months later from the combination of tumour progression and thromboembolic complications.

### Samples

Brain tumour biopsies were fixed in 10% formalin and embedded in paraffin wax from which 4-µm-thick sections were cut and stained with haematoxylin and eosin (H&E). The diagnosis of glioblastoma multiforme (WHO 9440/3) was made by light microscopical examination of H&E-stained sections. Glioblastoma was defined by WHO-2016 criteria of an infiltrative astrocytoma with proliferative activity, necrosis and/or microvascular proliferation.

To determine the *IDH1* status by immunohistochemistry, the 4-µm-thick sections were dried at 60 °C for 2 h and further processed on a Bond Max (Leica). After a 60-min pre-treatment with cell conditioner 2 (pH 6) the slides were incubated with 1:80 diluted H09 anti-*IDH1* R132H antibody (Dianova, Hamburg, Germany) at room temperature for 30 min. A standard 3,3’-diaminobenzidine (DAB) detection kit was used for chromogenic detection. The common *IDH1* mutation (R132H) was not detected.

To determine the *MGMT* promoter methylation, H&E-stained slides were reviewed and neoplastic cell-rich tissue was dissected from consecutive unstained sections. DNA was extracted from the dissected tissue using the QIAamp DNA FFPE Tissue Kit (Qiagen) and was bisulphite-converted using the EpiTect Bisulphite Kit (Qiagen). The *MGMT* promoter methylation was determined by pyrosequencing of four CpG sites (CpGs 76-79) in exon 1 of the *MGMT* gene using the CE-Marked therascreen *MGMT* Pyro Kit on a Pyromark Q24 System (Qiagen). Significant levels of *MGMT* promoter methylation were not detected.

To determine the tumour cell purity of the samples, H&E-stained slides were reviewed and cell counting was performed on multiple high-powered fields. The tumour cell purity of both the tumour and its margin was assessed by a pathologist prior to molecular profiling. No tumour cells were detected in the margins and the tumour was estimated to consist of 60% tumour cells.

### DNA and RNA extraction for sequencing

Each tumour and margin sample was frozen to −80 °C immediately on collection. The frozen samples were then defrosted and a 50 mg portion taken for DNA/RNA extraction. The samples were homogenised and then divided for DNA and RNA extraction. DNA extraction of both tissues and blood was performed using the QIAGEN DNeasy Blood and Tissue Kit, RNA extraction was performed using the QIAGEN RNeasy Mini Kit. Quantification was performed using the Qubit quantification assay. The tumour/normal pair used in this study was the sample judged most pure by comparison with blood DNA using whole genome sequencing.

### PCR-free oxBS and BS library preparation and sequencing

PCR-free ReBuilT libraries for bisulfite sequencing were generated following our previously published method.^12^ For the generation of PCR-free libraries for oxBS, libraries were oxidised (TrueMethyl Kit from CEGX) prior to bisulfite treatment. In brief, 350 and 175 ng of sonicated DNA from tumour and margin respectively were end repaired and dA-tailed before ligation of customized adapter pair. After (oxidation)-bisulfite treatment, degraded fragments were recovered through primer extension before dA-tailing and second adapter ligation. Libraries were subsequently immobilized on streptavidin coated magnetic beads, washed with binding buffer, eluted with 50 mM NaOH at 60 °C for 15 min. BS and oxBS-seq libraries were sequenced on an Illumina HiSeq 2500 platform, V4 chemistry, 2x101+7 index cycles.

### RNA-seq library preparation and sequencing

Libraries of the tumour and margin samples were prepared using TruSeq RNA Access kit (Illumina), using an input of 40 and 20 ng respectively. The libraries were sequenced for 2 × 75 cycles on HiSeq 2500 in Rapid mode achieving an average coding coverage of 108x (F2), 136X (F3), 277x (M1), 272x (M3).

### Computational analysis

#### Code availability

Details of the analysis are available in the manuscript's GitHub repository (https://github.com/sblab-bioinformatics/epigenetics-of-glioblastoma).

#### DNA-seq data analysis

Libraries were sequenced on an Illumina HiSeq 2500 platform. Alignment to the human reference genome GRCh37 and quality control was performed using Isaac.^36^ Identification of somatic SNVs and small somatic indels (<50 bp) was performed by Strelka.^37^ Large copy number variants and structural variants were respectively called with Canvas^38^ and Manta.^39^ The full workflow can be found in the Tumour Normal application of the Illumina BaseSpace platform (http://support.illumina.com/content/dam/illumina-support/help/BaseSpace_App_TumorNormal_v2/tumor-normal-v2-help.htm).

#### BS and oxBS-seq data analysis

Raw reads were trimmed to remove adapter sequences ligated to the 3'-end using cutadapt^40^ and aligned to the human reference genome hg19 using bwameth,^41^ a wrapper around bwa-mem.^42^

#### RNA-seq data analysis

As above, reads were trimmed to remove adapter sequences using cutadapt and aligned to reference transcripts obtained from Ensembl^43^ and quantified using kallisto.^44^

#### Tumour purity estimation

The copy number variant analysis using Canvas^38^ provided us with an estimate of 71% purity of the tumour sample.^45^ This software calculates coverage and tumour SNV allele frequencies at heterozygous germline positions along the genome. It then assigns copy numbers per genomic regions and infers genome-wide ploidy and purity by fitting the data to expected models for each copy number state given purity and diploid coverage level combinations. Purity is then derived from the best fitting model.

### Data availability

Data are available in the ArrayExpress database (www.ebi.ac.uk/arrayexpress) under accession number E-MTAB-5171

## Electronic supplementary material

Supplementary Figure 1

Supplementary Figure 2

Supplementary Figure 3

Supplementary Table 1

Supplementary Table 2

Supplementary information
