# Supplementary figures and images for: Base resolution maps reveal the importance of 5-hydroxymethylcytosine in a human glioblastoma
Source: NPJ Genom Med. 2017 Mar 13;2:6. doi: 10.1038/s41525-017-0007-6 (PMC5677956; doi:10.1038/s41525-017-0007-6)

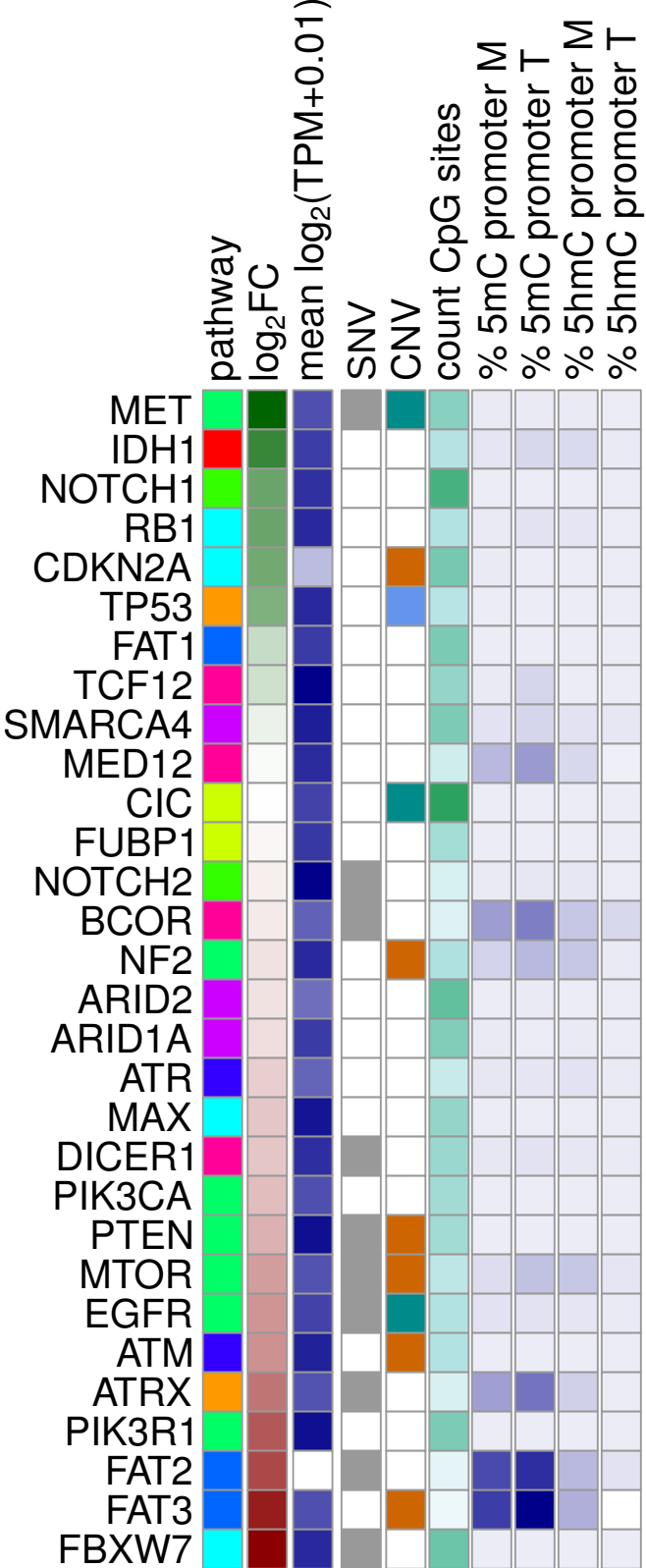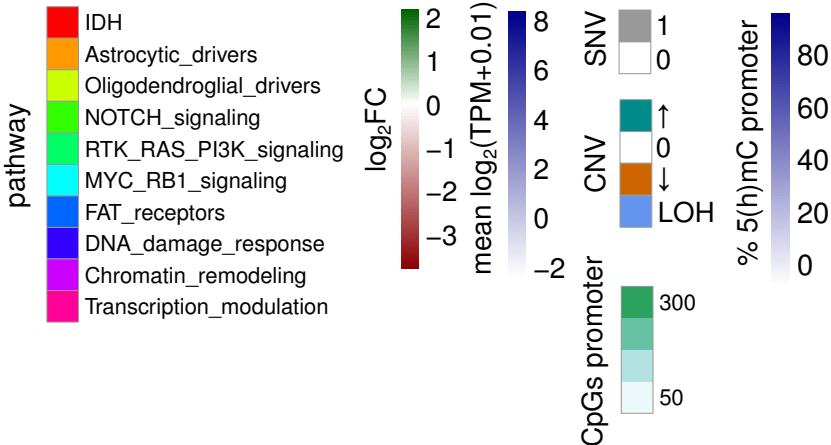

Supplement: Supplementary file 1 — Supplementary Figure 1 [file 41525_2017_7_MOESM1_ESM.pdf]

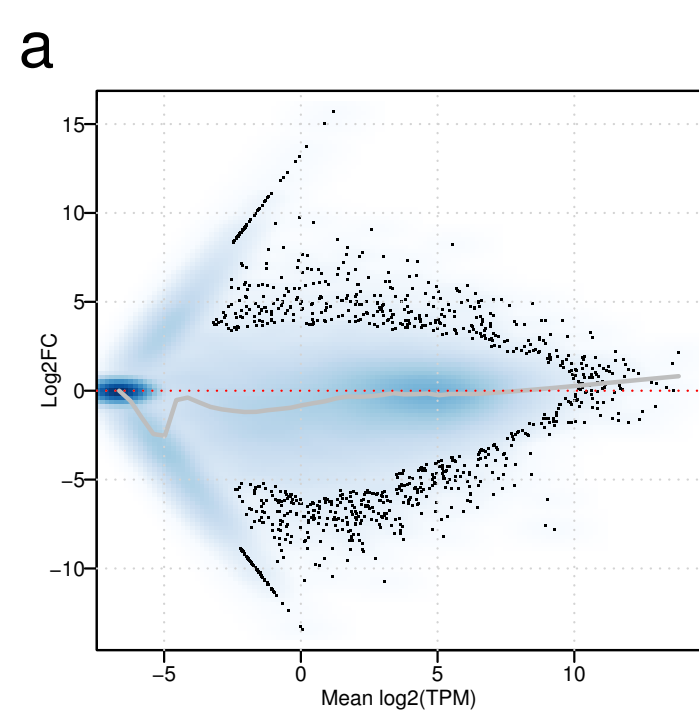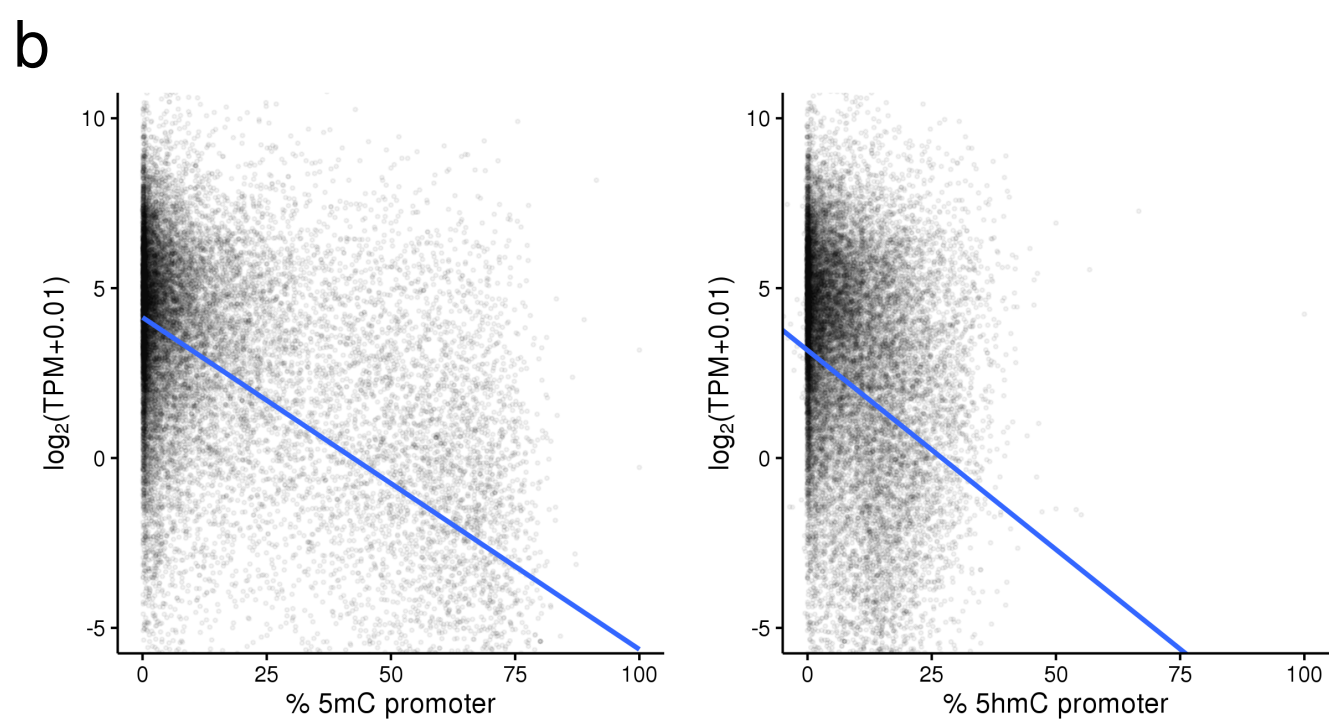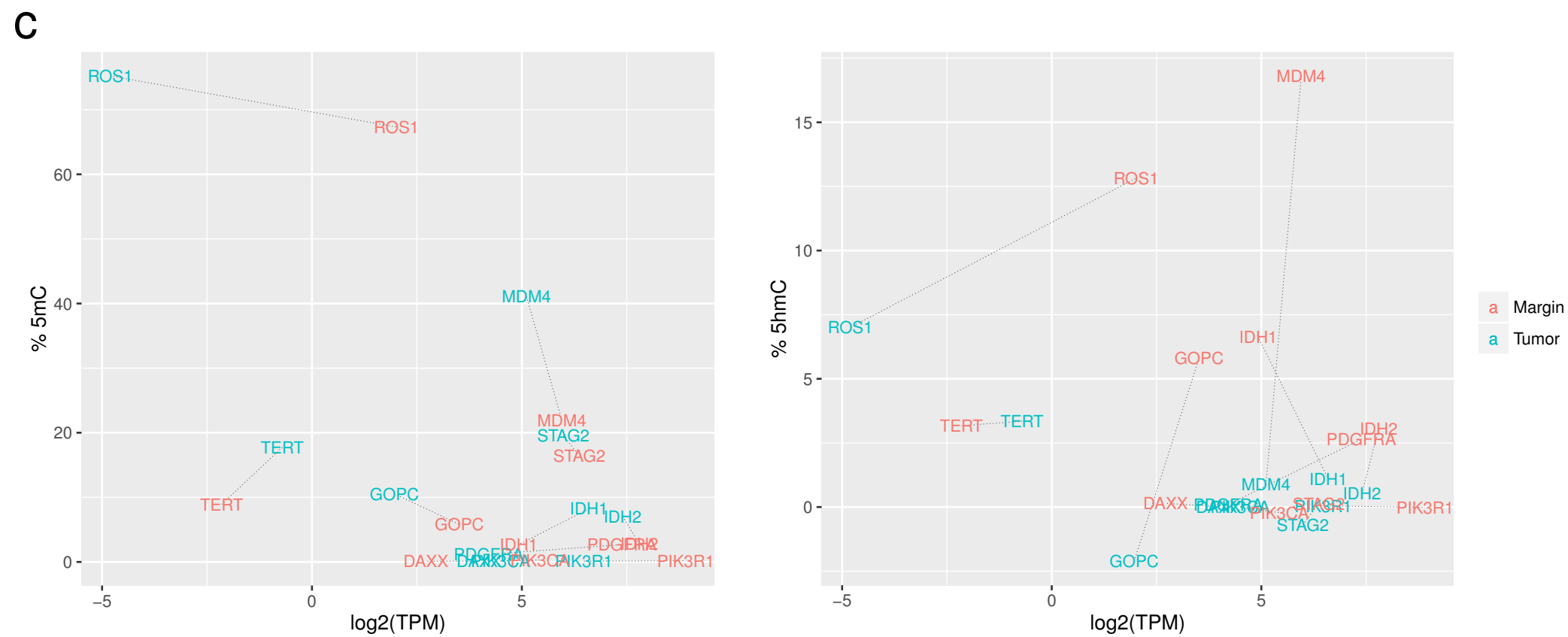

Supplement: Supplementary file 2 — Supplementary Figure 2 [file 41525_2017_7_MOESM2_ESM.pdf]

## 826 SNVs

Margin

|   | A   | C   | G   | T   |
|---|-----|-----|-----|-----|
| A | 0   | 29  | 132 | 34  |
| C | 51  | 0   | 42  | 150 |
| G | 135 | 41  | 0   | 43  |
| T | 31  | 111 | 27  | 0   |

## 7937 SNVs

Tumour

|   | A    | C   | G   | T    |
|---|------|-----|-----|------|
| A | 0    | 217 | 665 | 372  |
| C | 445  | 0   | 286 | 2074 |
| G | 1862 | 276 | 0   | 476  |
| T | 378  | 654 | 232 | 0    |

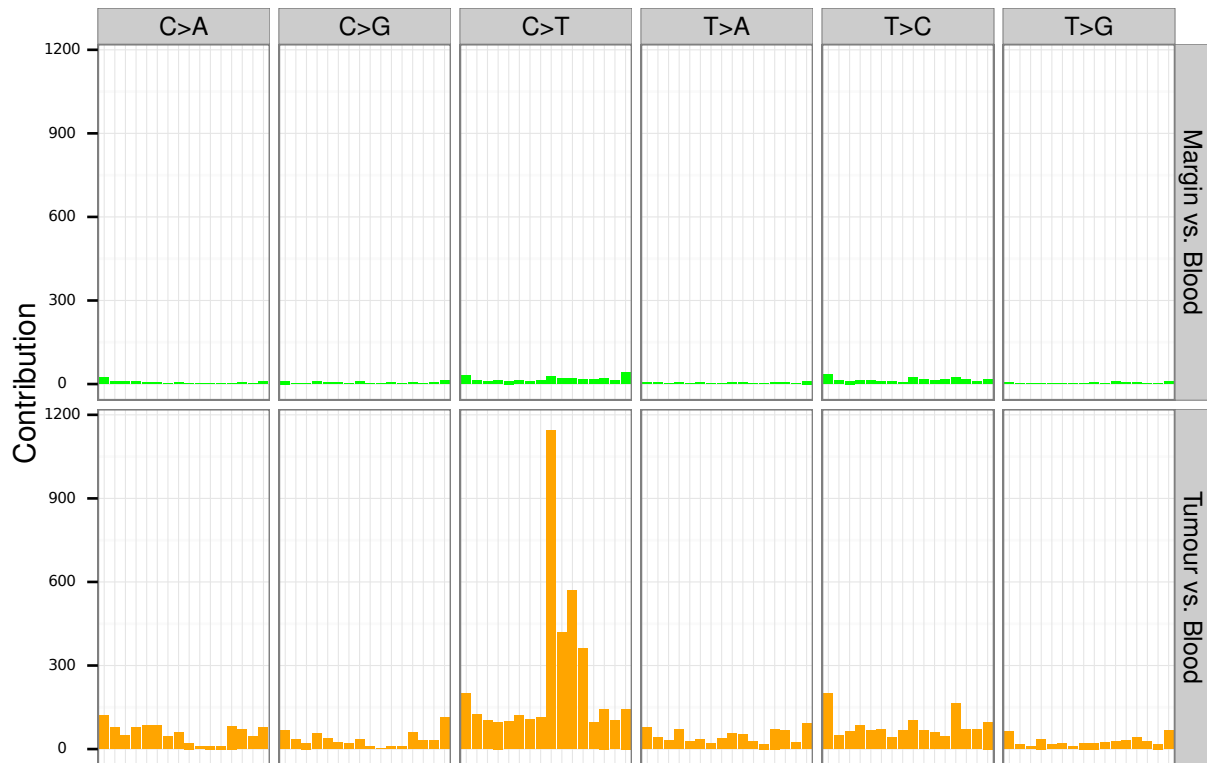

Supplement: Supplementary file 3 — Supplementary Figure 3 [file 41525_2017_7_MOESM3_ESM.pdf]
