# Supplementary material for: Base resolution maps reveal the importance of 5-hydroxymethylcytosine in a human glioblastoma
Source: NPJ Genom Med. 2017 Mar 13;2:6. doi: 10.1038/s41525-017-0007-6 (PMC5677956; doi:10.1038/s41525-017-0007-6)
Supplement: Supplementary file 4 — Supplementary Table 1 [file 41525_2017_7_MOESM4_ESM.docx]

**Supplementary Table 1**. Summary of sequencing statistics for each library. M8 and T3 refer to margin and tumour samples. BS and oxBS indicate (oxidative) bisulfite sequencing.
