# Supplementary material for: Base resolution maps reveal the importance of 5-hydroxymethylcytosine in a human glioblastoma
Source: NPJ Genom Med. 2017 Mar 13;2:6. doi: 10.1038/s41525-017-0007-6 (PMC5677956; doi:10.1038/s41525-017-0007-6)
Supplement: Supplementary file 5 — Supplementary Table 2 [file 41525_2017_7_MOESM5_ESM.docx]

**Supplementary Table 2**. Conversion rates in each library, separated by sequence and context.

| **Context** | **library_id** | **% 5(h)mC** | **% not CT** |
| --- | --- | --- | --- |
| CG | ear042_M8BS | 71.76 | 0.66 |
| CHG | ear042_M8BS | 1.37 | 0.39 |
| CHH | ear042_M8BS | 1.58 | 0.40 |
| CG | ear043_M8oxBS | 53.77 | 0.87 |
| CHG | ear043_M8oxBS | 1.05 | 0.57 |
| CHH | ear043_M8oxBS | 1.23 | 0.59 |
| CG | ear044_T3BS | 65.00 | 0.75 |
| CHG | ear044_T3BS | 0.20 | 0.47 |
| CHH | ear044_T3BS | 0.19 | 0.47 |
| CG | ear045_T3oxBS | 63.46 | 0.89 |
| CHG | ear045_T3oxBS | 0.19 | 0.61 |
| CHH | ear045_T3oxBS | 0.18 | 0.61 |
