# Supplementary material for: Base resolution maps reveal the importance of 5-hydroxymethylcytosine in a human glioblastoma
Source: NPJ Genom Med. 2017 Mar 13;2:6. doi: 10.1038/s41525-017-0007-6 (PMC5677956; doi:10.1038/s41525-017-0007-6)
Supplement: Supplementary file 6 — Supplementary information [file 41525_2017_7_MOESM6_ESM.docx]

**Supplementary Figure 1** Summary of the molecular details of the genes involved in high-grade gliomas as described in previous literature^24^. Differential transcript levels between tumour and margin (log_2_FC where FC = (TPM_tumour_ + 0.01)/(TPM_margin_ + 0.01)) and mean transcript levels ((log_2_(TPM_tumour_+0.01) + (log_2_(TPM_margin_+0.01))/2), SNVs (1: presence and 0: absence), CNVs (↑: gain of copies, 0: diploid and ↓: loss of copies and LOH: loss of heterozygosity), promoter CpG counts, and promoter 5mC and 5hmC levels (%) in margin (M) and tumour (T), which are colour-coded as shown in the legend.

**Supplementary Figure 2** Link between promoter 5mC/5hmC levels and gene expression. **(a)** Differential transcript levels between tumour and margin in vertical axis and mean transcript levels in horizontal axis – see **Fig. 2a** and **Supplementary Fig. 1** for details. **(b)** When 5mC and 5hmC levels are considered independently they inversely correlate with transcript levels. **(c)** Changes in transcript levels as a function of 5mC (left) and 5hmC (right) levels between margin and tumour for eleven genes with mutations associated to glioblastoma as defined by COSMIC^45^.

**Supplementary Figure 3** Mutational landscape of the blood and margin samples (tumour landscape is displayed in **Fig. 3a,b**).

**Supplementary Table 1** Summary of sequencing statistics for each library. M8 and T3 refer to margin and tumour samples.

**Supplementary Table 2** Conversion rates in each library, separated by sequence and context.
